# Heart Rate Variability, Autonomic Reactivity, and Emotion Regulation during Sadness Induction in Somatic Symptom Disorder

**DOI:** 10.1007/s12529-023-10238-2

**Published:** 2023-10-31

**Authors:** Laura Krempel, Johannes Stricker, Alexandra Martin

**Affiliations:** Department of Clinical Psychology and Psychotherapy, Institute of Psychology, School of Human and Social Sciences, University of Wuppertal, Gaußstraße 20, Wuppertal, 42119 Germany

**Keywords:** Autonomic reactivity, Emotion induction, Emotion regulation, Heart rate variability, Somatic symptom disorder

## Abstract

**Background:**

Preliminary evidence suggests altered heart rate variability (HRV) and impaired emotion regulation (ER) in somatic symptom disorder (SSD). Moreover, HRV can be considered an index of ER. Yet, to date, research on HRV and emotional reactivity in SSD is scarce and findings are inconsistent. Thus, this study aimed to examine ER differences, HRV at rest, and in response to emotion induction in persons with SSD compared to controls.

**Methods:**

The sample comprised 44 persons with SSD (DSM-5; 79.5% female, *M*_age_ = 45.7, *SD* = 14.7) and 41 persons without SSD (non-SSD; 78% female, *M*_age_ = 44.2, *SD* = 14.7). We assessed the participants’ somatic symptom severity, ER, and control variables (e.g., depressive symptoms). Frequency and time domain HRV by ECG and subjective emotional states were measured at rest, under sadness induction, and during recovery periods. We evaluated baseline between-group differences with *t*-tests, and HRV and emotional reactivity and recovery with repeated measures ANOVAs.

**Results:**

We found no significant differences in resting state HRV between persons with and without SSD. Regarding reactivity and recovery, SSD group showed lower reactivity in SDNN (standard deviation of NN interval) than non-SSD group. Moreover, SSD group reported more maladaptive ER techniques (e.g. rumination) and a higher effort to regulate their emotions during the experiment than non-SSD group.

**Conclusions:**

The study indicated impaired ER in persons with SSD. This finding showed more clearly in self-report than in HRV. Further research on HRV reactivity including tasks evoking other negative emotions in persons with SSD is required.

**Supplementary Information:**

The online version contains supplementary material available at 10.1007/s12529-023-10238-2.

## Introduction

Somatic symptom disorder (SSD) is characterized by one or more somatic symptoms accompanied by psychological distress, leading to impairment and suffering in daily life (see DSM-5; [1]). It replaces the former diagnoses of somatoform disorders and overlaps with several functional somatic syndromes (FSS) and pain conditions [2]. This disorder is characterized by high prevalence rates (e.g. 8.9% in Germany, [3]) and chronicity. However, little is known about the underlying mechanisms of SSD. As insights into psychopathological processes are vital for informing treatment, this study sought to investigate the role of emotion regulation (ER) and autonomic imbalance in SSD.

Preliminary findings suggest a deficit in ER as an important maintaining and aggravating factor in SSD [4]. Specifically, SSD has been associated with reduced emotional awareness, rigid emotional attention, and altered habitual ER strategy selection [5, 6]. Previous research shows that persons with SSD use more maladaptive (e.g., rumination) and fewer adaptive ER strategies (e.g. reappraisal) than persons without SSD [5–7]. Whereas maladaptive ER contributes to worsening of somatic symptoms [8], adaptive ER may reduce pain perceptions [9, 10]. Due to the impairments in ER, reactivity to emotional stimuli might be more intense and effortful in persons with SSD [6, 11]. So far, research into this question using experimental emotion induction tasks has yielded inconsistent findings [6]. For example, after presenting mood-inducing film clips, one study found higher arousal and more negative affect in persons with fibromyalgia than in healthy controls [11]. Yet, other studies found no differences in response to emotion induction between persons with and without SSD [12, 13].

Heart rate variability (HRV) is often used as a proxy for the regulatory processes of the autonomic nervous system (ANS) and is assumed as a physiological marker for ER [14, 15]. Therefore, resting state HRV and phasic HRV during challenging tasks are crucial for determining ER deficits [16]. According to the Model of Neurovisceral Integration, lower HRV, especially reduced vagal tone, is associated with self-regulation problems and reduced ER capacity [17, 18]. The modulation of parasympathetic activity by the prefrontal cortex through inhibition of sympathoexcitatory mechanisms is central to this association. Thus, low HRV results from withdrawn inhibition processes and the emergence of sympathetic activity, and is viewed as a transdiagnostic marker for psychopathology [17–19].

To, date, initial evidence suggests autonomic imbalance in form of reduced HRV in somatoform disorders, pain conditions, and FSS [20, 21]. These disorders are highly similar to SSD. Thus, reduced HRV should also be present in persons with SSD. A recent meta-analysis revealed resting-state HRV to be lower in SSD compared to healthy controls [22]. However, this meta-analysis primarily included studies on former diagnoses of somatoform disorders, pain conditions, and FSS. Specifics of HRV in SSD (based on the new DSM-5 criteria) have seldom been studied. A few existing studies suggest reduced parasympathetic activity in SSD, but there is no consistent evidence for the different HRV parameters [23–25]. Whereas some studies found lower levels in time-domain parameters [24], others found lower levels in frequency-domain parameters [24, 25] or no differences in persons with SSD compared to healthy controls [23–25].

Regarding phasic HRV during emotionally challenging tasks, recent studies showed that HRV reactivity is characterized by HRV decrease during sad film clips in healthy subjects [26, 27]. In contrast, healthy individuals with reduced vagal tone and ER deficits showed lower HRV reactivity and delayed vagal control recovery [26, 28]. Concerning SSD, most research examined HRV during physical tasks (e.g., cold pressor test) but not during psychological challenges (e.g., Stroop task). There is initial evidence for lower HRV reactivity in SSD in both task types compared to healthy controls [22]. However, other studies showed no abnormalities in HRV reactivity in fibromyalgia and chronic fatigue syndrome compared to healthy subjects [11, 29]. Particularly studies using psychological challenges indicated lower HRV reactivity in SSD compared to healthy controls but results for the different parameters are, again, inconsistent [24, 30–32]. In summary, HRV abnormalities and emotional reactivity in SSD (based on the new DSM-5 criteria) have seldom been studied. The existing studies primarily investigate resting state HRV and reactivity and seldom HRV recovery in experimental designs. Preliminary findings do not provide a clear picture, and studies involving emotional reactivity are still lacking. Nevertheless, based on the preliminary findings on ER deficits in SSD, it can be assumed that altered HRV response occurs in emotionally demanding situations.

This study examined ER in reaction to sadness-inducing films on the subjective (self-report) and objective (HRV) level in persons with and without SSD. Film clips were used because it is a valid method for inducing emotion [27, 33]. Sadness-inducing films show a clearer triggering of sadness (emotion-specifity) while induction of fear additionally often trigger disgust and surprise [27, 33]. Using an experimental design to assess HRV reactivity and recovery during emotional challenges provides essential insight into the relationship between autonomic activity and ER in SSD. Specifically, this study had three central aims. First, we aimed to compare baseline ER strategies and resting state HRV between persons with and without SSD. We expected stronger maladaptive ER techniques and fewer adaptive ER techniques (assessed via self-report) (Hypothesis 1) and lower HRV [lower root mean square of successive differences (RMSSD), lower Standard Deviation of the NN Interval (SDNN), lower low-frequency power (LF), lower high-frequency power (HF)] at rest in persons with SSD (Hypothesis 2). Second, we aimed to examine HRV and subjective emotional states during (HRV reactivity) and after (HRV recovery) sadness-inducing film clips. We expected a lower HRV (lower RMSSD, lower SDNN, lower LF, lower HF) and emotional (lower valence and energy, higher tension and arousal) reactivity during (Hypothesis 3) and delayed recovery after (Hypothesis 4) a sadness-inducing film clip in persons with SSD than in persons without SSD. Third, we aimed to explore the associations of psychopathological variables, primarily somatic symptom burden, with HRV. According to the Model of Neurovisceral Integration [18] we hypothesized that lower HRV is associated with more pronounced psychopathology (Hypothesis 5).

## Methods

### Procedures

The study was conducted at the Department of Clinical Psychology and Psychotherapy of a University in Germany. The local ethics committee approved this study (MS/BBL 190,327). The sample comprised participants diagnosed with SSD and controls without SSD (non-SSD). SSD participants were part of a pilot randomized controlled trial for HRV biofeedback (https://drks.de/search/de/trial/DRKS00017099) [34]. After the experiment, intervention followed. Recruitment and diagnostic procedures of the SSD group took place between March 2019 and February 2021 and have been described in detail elsewhere [34]. Controls were matched to the SSD group in terms of age and gender and were recruited between November 2020 and August 2021 through flyers, the university’s newsletter, and social media. Exclusion criteria for all participants were age younger than 18 or older than 65 years, pacemaker, no stable medication within the last four weeks, acute heart disease (e.g., myocarditis), BMI (Body Mass Index) > 30, diabetes, rheumatism, substance use disorder and psychotic symptoms in the past. For controls, additional exclusion criteria were physical illness (e.g. migraine), a score in the Patient Health Questionnaire 15 (PHQ-15, [35]) ≥ 9, and a score in the Somatic Symptom Disorder Scale (SSD-12, [36]) ≥ 23 [37]. All participants in the control group were first screened for age, PHQ-15, and SSD-12 scores via online questionnaires. In the next step, further exclusion criteria (e.g. medication, physical illness) were screened and a structured interview for DSM-5 for SSD diagnosis [37] was conducted in a telephone call by trained psychologists. Participants were informed not to engage in vigorous physical activity (e.g., doing exercises or bicycling to the examination) beginning the evening before the assessment. All eligible participants provided written informed consent and were invited for the assessment.

### Heart Rate Variability

HRV was measured with an electrocardiogram recording with 1024 Hz sampling by attaching electrodes on the right or left clavicle, under the left or right costal arch (active electrodes), and on the right arm (ground electrode). Respiration was measured with 32 samples per second with strain gauges around the chest and abdomen. After an adaptation period of five minutes, resting state HRV was measured for five minutes (baseline). Participants sat on a chair while viewing a blank screen with a fixation cross. They were instructed to breathe normally (with no indication to relax). Afterward, the brief film clip was administered (emotion induction; reactivity). The resting-state HRV was recorded again after the film clip for five minutes (recovery).

Electrocardiogram signals were assessed with the NEXUS-4 through Biotrace software (Mindmedia) and analyzed with Kubios software [38]. The interbeat intervals of the electrocardiograms were exported for the three periods (baseline, emotion induction, recovery) from Biotrace and imported into Kubios for further analyses. After the automatic artifact correction algorithm of Kubios [39], we carefully checked visually for artifacts. A fast Fourier transformation was performed and the power was quantified in two frequency-domain measures: low-frequency power (LF, 0.04–0.15 Hz), and high-frequency power (HF, 0.15–0.40 Hz). HF is associated with parasympathetic activity, whereas LF captures parasympathetic and sympathetic activity [40]. We also calculated time-domain-based HRV parameters: SDNN and RMSSD. SDNN represents the overall variability, and RMSSD represents the vagally mediated changes in HRV [40]. For the baseline resting state measure, we used the entire five minutes interval. Because the emotion induction lasted only two minutes, the measurement periods for the reactivity and recovery analyses were adjusted (the last two minutes of the baseline period and emotion induction period, and the first two minutes of the recovery period).

### Psychometric Measurements

#### State Valence and Arousal

We assessed state valence and arousal with the Self-Assessment Manikin (SAM, [41]). This instrument is a picture-oriented nine-point scale that contains five pictures of a manikin on each of its two subscales. Higher scores indicate higher (positive) valence and higher arousal. It was assessed before, during, and after the emotion induction in paper-pencil format.

#### Subjective State Tension, Energy, and Sadness

We used visual analog scales (VAS, [42]) to assess subjective emotional response before, during, and after the emotion induction on three dimensions: tension (“I feel tense.“), energy (“I feel energized and active.”), sadness (“I feel sad.”). The VAS had a length of ten centimeters ranging from “not at all” (0) to “absolutely” (100; one millimeter representing one point). Participants were instructed to rate their present feeling for every dimension by making a stroke on the respective line.

#### Emotion Regulation

Emotion regulation was measured with the Heidelberg Form for Emotion Regulation Strategies (HFERST, [43]) before the experiment. It assesses adaptive (reappraisal, social support, problem-solving, acceptance) and maladaptive (rumination, experience suppression, expressive suppression, avoidance) ER strategies over the last four weeks with 28 items on a 5-point Likert scale. Internal consistencies for the subscales ranged from *α* = 0.77 to *α* = 0.88.

#### Psychopathology

Participants completed several instruments assessing psychopathology before the emotion induction. The Screening for Somatoform Disorders (SOMS-7T, [44]) is a 53-item self-report questionnaire that assesses somatic symptom severity in the last week on a 5-point Likert scale (not at all to very strong, sum score 0–208). The German SOMS-7T has frequently displayed good reliability and validity [44]. In this study, the internal consistency was *α* = 0.94.

The Somatic Symptom Disorder 12 Scale (SSD-12, [36]) assesses the psychobehavioral aspects of SSD according to the B-criteria of DSM-5 with 12 items on a 5-point Likert scale (“never” to “very often”, sum score 0–48) and satisfactory internal consistency in this study (*α* = 0.95).

The Patient Health Questionnaire 9 (PHQ-9, [45]) measures the severity of depression through nine items on a 4-point Likert scale. The sum score ranges from 0 to 27 with higher scores indicating a higher severity degree of depression. In this study, internal consistency was *α* = 0.89. Anxiety was measured with the Generalized Anxiety Disorder Scale (GAD-7, [46]) through 7 items on a 4-point Likert scale (sum score 0–21) with higher scores indicating a higher degree of anxiety. Internal consistency was good (*α* = 0.89).

### Emotion Induction

We induced negative affect by presenting one of two brief film clips without sound. Film clip assignments were randomized within each group. We used two different film clips to control for specific film effects. Thus, using two film clips provides a broader base for induction and allows more broadly generalizing the findings for the emotion of sadness. The films contained either a sequence of the movie “Walk the line” (the USA, 2005) with a duration of 2:14 min or a sequence of the movie “Oscar et la dame rose” (France, 2009) with a duration of 3:06 min. Both clips were validated to induce sadness [33]. In our study, *U*-tests for VAS sadness revealed a significant increase in sadness from baseline for both groups (SSD: *z* = -3.23, *p* = .001; non-SSD: *z* = -5.21, *p* < .001). Film clips did not differ in their induced sadness level (*z* = -1.28, *p* =. 20). Therefore, negative emotion induction was successful.

### Experimental Procedure

**Fig. 1:**
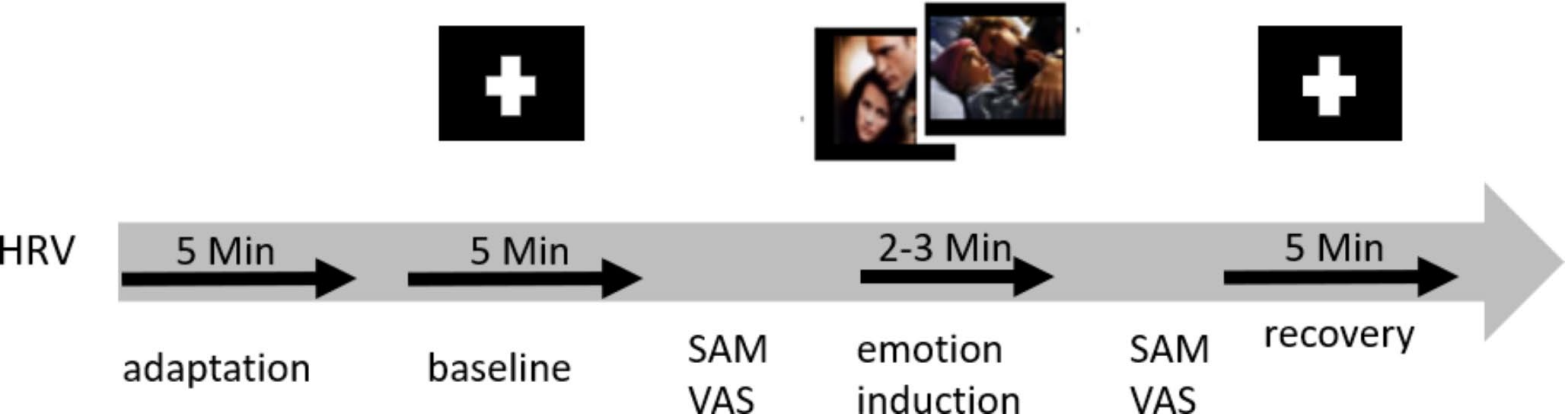
Procedure of the experiment HRV = heart rate variability; SAM = self-assessment manikin; VAS = visual analogue scale

The experimental procedure is illustrated in Fig. 1. After completing a psychometric test battery and an adaptation phase of five minutes, participants’ baseline HRV was assessed for five minutes. During this period, participants looked at a blank screen with a fixation cross (baseline). Participants then completed the SAM and VAS. Afterward, participants viewed a film clip while HRV was recorded (emotion induction) and completed the SAM and VAS. Then, five minutes of rest followed (recovery), and participants completed the SAM and VAS again. Participants were asked not to do strong physical activity on the day of the assessment and not to consume nicotine, caffeine, or alcohol four hours before.

### Statistical Analysis

We conducted the analyses with IBM SPSS 28 (SPSS Inc, Chicago, IL). Complete data sets for the HRV, baseline questionnaires, and VAS were available for *n* = 43 SSD and *n* = 41 non-SSD participants; for the SAM *n* = 42 for SSD and *n* = 39 for non-SSD. We excluded individuals with cardiovascular medication from HRV analyses (SSD: *n* = 4; non-SSD: *n* = 2). We identified three outliers in SAM and VAS data with boxplots (three standard deviations from the mean). The normal distribution of all data was checked with the Shapiro-Wilk test. Group differences at baseline in demographic, health, and psychological variables were tested with *t*-tests for continuous and *χ²*-test for categorical variables. For non-normal distributed questionnaire data, we used *U*- tests. For HRV parameters with non-normal distribution, data were transformed with a natural logarithm. To identify emotion regulation strategy (HFERST) differences between groups (SSD/non-SSD), we conducted a multivariate analysis of variance (MANOVA) with post hoc univariate analyses of variance (ANOVAs). For resting activity at baseline, HRV parameters (lnRMSSD, lnSDNN, lnLF, lnHF) were compared between SSD and non-SSD groups by using ANCOVAs adjusting for age. For evaluating HRV reactivity and recovery and subjective states, we used 2 (group: SSD, non-SSD) x 3 (time: baseline, emotion induction, recovery) mixed ANCOVAs adjusting for age and Bonferroni corrected post hoc *t*-tests, adjusting the alpha level to 0.01. We conducted a sensitivity analysis in which we excluded male participants from the HRV analyses, as gender may interfere with HRV [47]. Finally, we computed the bivariate correlations between the HRV parameters at baseline and psychological variables. We computed bivariate correlations between HRV parameters and possible influencing variables (age, gender, tobacco consumption, medical condition, see Supplementary Material Table S1).

## Results

### Demographic Characteristics and Psychopathology

Table 1 shows the demographic data, health-related and psychological variables. The mean age of the SSD group was 45.4 years (SD = 14.8) and that of the control group was 44.1 years (SD = 14.7). Both groups did not differ in any sociodemographic variable (all *p*s ≥ 0.08) except for tobacco consumption. SSD participants were more likely to smoke than non-SSD participants (*χ²*(1) = 5.45, *p* = .02).

**Table 1:** Demographic, health, and psychological variables data for persons with and without Somatic Symptom Disorder

	total *(n* = 84)			SSD (*n* = 43)			Non-SSD (*n* = 41)		
	*M/N*	*SD/%*		*M/N*	*SD/%*		*M/N*	*SD/%*	test statistics
**age** (*M*/*SD*)	44.77	14.67		45.35	14.78		44.17	14.72	*U =* 860, *p* = .85
**gender (female)** (*N*/*%*)	67	78.82		35	79.55		32	78.05	*χ²*(1) = 0.01, *p* = .91
**BMI** (*M*/*SD*)	23.70	3.05		23.97	3.40		23.55	2.75	*U =* 854, *p* = .80
**partnership/married** (*N*/*%*)	52	61.9		27	62.8		25	60.9	*χ²*(1) = 0.03, *p* = .86
**employment status** (*N*/*%*)									*χ²*(2) = 5.19, *p* = .08
employed	60	71.4		26	60.4		34	82.9	
in training	10	11.9		7	16.3		3	7.3	
unemployed/retired	14	16.7		10	23.3		4	9.8	
**tobacco consumption** (yes) (*N*/%)	17	20.2		13	30.2		4	9.8	*χ²*(1) = 5.45, *p* = .02*
**cardiac medication** (yes) (*N*/*%*)	6	7.1		4	9.3		2	4.9	*χ²*(1) = 0.62, *p* = .43
**psychopharmacological medication** (yes) (*N*/*%*)	7	8.3		6	7.1		1	1.2	*χ²*(1) = 3.6, *p* = .06
**comorbid somatic disease** (yes) (*N*/*%*)	26	30.9		10	23		16	39	*χ²*(1) = 2.4, *p* = .12
**SOMS-7** (*M*/*SD*)	19.22	21.7		32.05	20.84		4.14	3.91	*U =* 95, *p* = .001**
**PHQ-15** (*M*/*SD*)	8.13	5.88		13.00	4.11		3.27	2.19	*t*(82) = -13.38, *p* = .001**
**SSD-12** (*M*/*SD*)	16.81	12.99		27.42	8.55		5.68	5.09	*U =* 41, *p* = .001**
**PHQ-9** (*M*/*SD*)	5.86	5.88		9.26	5.81		2.29	1.76	*U =* 200, *p* < .001***
**GAD-7** (*M*/*SD*)	5.15	4.84		8.39	4.62		1.76	1.76	*U =* 101, *p* = .001**

*Note.* SSD = somatic symptom disorder; BMI = body mass index; SOMS- 7 = Screening for Somatoform Disorders; PHQ-15 = Patient Health Questionnaire 15; SSD-12 = Somatic Symptom Disorder Scale- 12; PHQ-9 = Patient Health Questionnaire; GAD-7 = Generalized Anxiety Disorder Scale

* p < .05

** p < .01

*** p < .001

SSD participants reported a mean symptom duration of 9.14 years (SD = 9.70). Of those with SSD, 77% had predominantly pain (DSM-5 specifier) and 52.3% showed a comorbid mental disorder. SSD group had significantly higher scores on all psychopathological variables (PHQ-15, SSD-12, PHQ-9, GAD-7) than the non-SSD group (all *p’s* < 0.001). Table S2 (Supplementary Material) shows an overview of medical conditions reported in our sample.

### Self-reported Emotion Regulation Strategies

The MANOVA for HFERST revealed a significant multivariate group effect (*F*(8, 76) = 10.21, *Λ* = 0.01, *p* < .001, *η*_*p*_*²* = 0.52; see Table 2). The SSD group displayed significantly higher rumination and expressive suppression and lower reappraisal and acceptance than the non-SSD group (4.5 > *F’s* < 32, *ps* < 0.05).

**Table 2:** Emotion regulation characteristics assessed with HFERST in participants with SSD and non- SSD

	SSD (*n* = 43)			Non-SSD (*n* = 41)		
Variable	*M*	*SD*		*M*	*SD*	ANOVA test statistics
rumination	3.75	0.91		2.76	0.93	*F*_g_(1, 82) = 20.48, *p* = .00**, *η*_*p*_*²* = 0.23
reappraisal	2.85	0.92		3.38	0.97	*F*_g_(1, 82) = 6.45, *p* = .01*, *η*_*p*_*²* = 0.07
acceptance	2.85	0.85		3.80	0.65	*F*_g_(1, 82) = 32.92, *p = .*00**, *η*_*p*_*²* = 0.29
problem solving	4.14	0.77		4.00	0.58	*F*_g_(1, 82) = 0.89, *p* = .35, *η*_*p*_*²* = 0.01
expressive suppression	3.19	1.07		2.75	0.77	*F*_g_(1, 82) = 4.56, *p* = .04*, *η*_*p*_*²* = 0.05
experience suppression	2.47	0.93		2.24	0.71	*F*_g_(1, 82) = 1.59, *p* = .21, *η*_*p*_*²* = 0.02
avoidance	3.12	0.96		2.81	0.85	*F*_g_(1, 82) = 2.46, *p* = .12, *η*_*p*_*²* = 0.03
social support	3.41	1.12		3.70	1.11	*F*_g_(1, 82) = 1.39, *p* = .24, *η*_*p*_*²* = 0.02

*Note.* SSD = somatic symptom disorder; HFERST = Heidelberg Form for Emotion Regulation Strategies

Score range HFERST: 1 (“never”) – 5 (”always”)

* p < .05

** p < .01

### Rest HRV at Baseline

There were no statistically significant differences between the SSD and non-SSD group in any HRV parameter (lnRMSSD, lnSDNN, lnLF, lnHF) at baseline, 0.09 ≤ *ps* ≤ 0.14; see Table 3). When excluding male individuals from the analyses, the above reported results did not change.

**Table 3:** Test statistics for the baseline HRV t-tests assessing differences between the SSD and non-SSD groups

Baseline HRV parameter	SSD			Non-SSD		ANCOVA test statistics
	*M*	*SD*		*M*	*SD*	
lnRMSSD	3.35	0.73		3.58	0.73	*F*_*g*_(1, 75) = 2.18, *p* = .14, *η*_*p*_*²* = 0.03
lnSDNN	3.46	0.63		3.69	0.63	*F*_*g*_(1, 75) = 3.03, *p* = .09, *η*_*p*_*²* = 0.04
lnLF	6.04	1.30		6.41	1.23	*F*_*g*_(1, 75) = 1.71, *p* = .20, *η*_*p*_*²* = 0.02
lnHF	5.74	1.49		6.29	1.57	*F*_*g*_(1, 75) = 2.82, *p* = .10, *η*_*p*_*²* = 0.04

*Note.* SSD = somatic symptom disorder; ln = natural logarithm; RMSSD = Root mean square of successive differences; SDNN = Standard Deviation of the NN Interval; LF = low frequency; HF = high frequency

All *p* values are calculated with the analysis of covariance (ANCOVA) after adjusting for age

### HRV and Emotional Reactivity and Recovery

#### HRV

A series of ANCOVAs revealed significant time effect and group x time effect for the lnSDNN only, (*p*s ≤ 0.05) indicating autonomic changes during the experiment (see Table 4 & Supplementary Material Figure S1).

**Table 4:** HRV at baseline, during the emotion induction and the recovery period for the SSD and non- SSD groups

	SSD (*n* = 39)			Non-SSD (*n* = 39)		
Variable	*M*	*SD*		*M*	*SD*	ANCOVA test statistics for each variable
**lnRMSSD**						
BL	3.27	0.72		3.52	0.76	*F*_t_(2, 150) = 0.77, *p* = .47, *η*_*p*_*²* = 0.32 *F*_g_(1, 75) = 1.28, *p* = .26, *η*_*p*_*²* = 0.02 *F*_int_(2, 150) = 2.61, *p* = .08, *η*_*p*_*²* = 0.03
EI	3.39	0.75		3.55	0.66	
REC	3.40	0.72		3.54	0.69	
**lnSDNN**						
BL	3.38	0.63		3.66	0.65	*F*_t_(2, 150) = 3.17, *p* = .045*, *η*_*p*_*²* = 0.04 *F*_g_(1, 75) = 2.36, *p* = .13, *η*_*p*_*²* = 0.03 *F*_int_(2, 150) = 3.33, *p* = .04*, *η*_*p*_*²* = 0.04
EI	3.41	0.61		3.56	0.55	
REC	3.52	0.61		3.66	0.60	
**lnLF**						
BL	5.86	1.34		6.41	1.44	*F*_t_(1.83, 137.55) = 2.88, *p* = .06, *η*_*p*_*²* = 0.04 *F*_g_(1, 75) = 1.75, *p* = .19, *η*_*p*_*²* = 0.02 *F*_int_(1.83, 137.55) = 1.82, *p* = .17, *η*_*p*_*²* = 0.02
EI	5.88	1.22		6.03	1.17	
REC	5.98	1.29		6.31	1.26	
**lnHF**						
BL	5.66	1.48		6.23	1.64	*F*_t_(1.75, 131.36) = 1.63, *p* = .20, *η*_*p*_*²* = 0.02 *F*_g_(1, 75) = 2.15, *p* = .15, *η*_*p*_*²* = 0.03 *F*_int_(1.75, 131.36) = 1.38, *p* = .25, *η*_*p*_*²* = 0.02
EI	5.87	1.43		6.20	1.38	
REC	5.76	1.44		6.22	1.56	

*Note.* SSD = somatic symptom disorder; ln = natural logarithm; RMSSD = Root mean square of successive differences; SDNN = Standard Deviation of the NN Interval; LF = low frequency; HF = high frequency; BL = baseline; EI = emotion induction; REC = recovery period

All p values are calculated with the analysis of covariance (ANCOVA) after adjusting for age

* *p* < .05

The SSD and non-SSD groups showed a differential reactivity and recovery in lnSDNN. As expected, the non-SSD group showed a decrease from baseline to emotion induction (*t*(38) = 2.00, *p* = .03, *d* = 0.32), and a significant increase from the emotion induction to the recovery period (*t*(38) = -2.50, *p* = .008, *d* = .-0.40). In contrast, the SSD group showed no significant change from baseline to emotion induction (*t*(38) = -0.93, *p* = .18, *d* = − 0.15) and from emotion induction to the recovery period (*t*(38) = -2.21, *p* = .02, *d* = − 0.35). The SSD group had an increase in lnSDNN from baseline to recovery period (*t*(38) = -3.70, *p* < .001, *d* = − 0.59) while the non-SSD group showed no significant difference (*t*(38) = 0.42, *p* = .42, *d* = 0.22). When excluding male individuals from the analyses, we did not find any significant effects (see Supplementary Material Table S3).

#### State Valence and Arousal

A series of ANOVAs indicated significant time effects for affect and arousal (*p*s < 0.001; see Table 5 & Supplementary Material Figure S2). For valence, there was also a significant group effect (*p*’s < 0.001). The SSD group showed significantly more negative valence levels across all three time points than the non-SSD group (*ps* ≤ 0.003). The groups did not differ in their reactivity and recovery concerning valence and arousal (decrease from baseline to emotion induction and an increase in recovery, *p*’s < 0.001). Table S4 shows the results for the reduced sample without individuals taking cardiovascular medication. Results did not change.

**Table 5:** Subjective ratings (SAM and VAS) at baseline, in the emotion induction phase, and at the recovery period for the SSD and non-SSD groups

	SSD (*n* = 43)			Non-SSD (*n* = 36)			
	*M*	*SD*		*M*	*SD*	ANOVA test statistics	
**valence (SAM)**							
BL	5.26	1.75		7.08	1.13	*F*_t_(1.65, 126) = 36.02, *p* < .001***, *η*_*p*_*²* = 0.32 *F*_g_(1, 77) = 25.95, *p* < .001***, *η*_*p*_*²* = 0.25 *F*_int_(1.65, 126) = 1.84, *p* = .17, *η*_*p*_*²* = 0.02	
EI	3.86	1.60		5.19	1.90		
REC	5.21	1.47		6.28	1.63		
**arousal (SAM)**							
BL	3.21	2.15		2.47	1.25	*F*_t_(2, 152) = 22.55, *p* < .001***, *η*_*p*_*²* = 0.23 *F*_g_(1, 76) = 1.09, *p* = .30, *η*_*p*_*²* = 0.01 *F*_int_(2, 152) = 1.68, *p* = .19, *η*_*p*_*²* = 0.02	
EI	3.98	1.91		3.75	1.86		
REC	2.62	1.40		2.58	1.65		
**tension (VAS)**							
BL	29.60	25.89		20.05	19.12	*F*_t_(2, 158) = 15.96, *p* < .001***, *η*_*p*_*²* = 0.17 *F*_g_(1, 79) = 4.36, *p* = .04*, *η*_*p*_*²* = 0.05 *F*_int_(2, 158) = 0.98, *p* = .38, *η*_*p*_*²* = 0.01	
EI	36.00	25.49		30.42	25.13		
REC	25.86	24.43		13.55	17.56		
**energy (VAS)**							
BL	30.88	28.29		41.79	24.45	*F*_t_(2, 152) = 6.00, *p* < .001***, *η*_*p*_*²* = 0.07 *F*_g_(1, 76) = 7.32, *p* = .008**, *η*_*p*_*²* = 0.09 *F*_int_(2, 152) = 1.33, *p* = .27, *η*_*p*_*²* = 0.02	
EI	24.30	21.81		37.45	26.37		
REC	21.23	21.96		38.58	27.63		

*Note.* SSD = somatic symptom disorder; VAS = visual analogue scale; SAM = self-assessment manikin; BL = baseline; EI = emotion induction; REC = recovery period

Score range SAM: 1–9; score range: VAS: 0–100

* *p* < .05

** *p* < .01

*** *p* < .001

#### State Tension and Energy

We found significant time (*p*s < 0.01) and group effects (*p*s < 0.05) for tension and energy (see Table 5 & Supplementary Material Figure S2). For tension reactivity, the SSD group showed no change from baseline to the emotion induction phase (*t*(42) = -1.66, *p* = .05, *d* = − 0.25) whereas the non-SSD group showed a significant increase, *t*(37) = -3.22, *p* = .001, *d* = − 0.52. The groups showed no differences in the change from the emotion induction to the recovery phase (SSD: *t*(42) = 2.94, *p* = .005, *d* = 0.45; non-SSD: *t*(37) = 5.43, *p* < .001, *d* = 0.88). The SSD group showed significantly lower energy level in the recovery period than the-SSD group (*t*(79) = -3.14, *p* = .001, *d* = − 0.70). Additionally, the SSD group reported significantly decreasing energy levels from baseline over emotion induction to recovery (*ps* ≤ 0.001). The non-SSD group showed no significant change in energy over the three measurement points (*ps* > 0.11). Table S4 shows the results for the reduced sample without individuals taking cardiovascular medication. The results did not change, except for tension (*p* = .09).

### Association of HRV and Psychopathology

We observed significant correlations between somatic symptom severity (SOMS-7) and lnSDNN (*ρ*= − 0.36, *p* = .02) and lnLF (*ρ*= − 0.39, *p* = .01) in SSD group, and between SOMS-7 and lnRMSSD (*ρ* = − 0.48, *p* = .002) and lnHF (*ρ* = 0.43, *p* = .007) in non-SSD group (see Table 6).

**Table 6:** Bivariate correlations (Spearman) between HRV parameters at rest and psychopathology variables for each group separately

Variable	Group	SOMS-7	PHQ-15	SSD-12	PHQ-9	GAD-7
lnSDNN	SSD	− 0.36*	− 0.36*	− 0.05	− 0.18	− 0.27
	Non-SSD	− 0.28	− 0.13	− 0.21	0.06	− 0.19
lnRMSSD	SSD	− 0.22	− 0.26	− 0.03	− 0.10	− 0.23
	Non-SSD	− 0.48**	− 0.12	− 0.21	0.03	− 0.11
lnLF	SSD	− 0.39*	− 0.40*	− 0.05	− 0.16	− 0.31
	Non-SSD	− 0.00	− 0.13	− 0.19	0.14	− 0.16
lnHF	SSD	− 0.21	− 0.23	− 0.02	− 0.10	− 0.22
	Non-SSD	− 0.43**	0.02	− 0.16	0.06	− 0.08

*Note.* SSD = somatic symptom disorder; SOMS-7 = Screening for Somatoform Disorders; PHQ-15 = Patient Health Questionnaire 15; SSD-12 = Somatic Symptom Disorder Scale- 12; PHQ-9 = Patient Health Questionnaire; GAD-7 = Generalized Anxiety Disorder Scale; ln = natural logarithm; SDNN = standard deviation of the NN interval; RMSSD = root mean square of successive differences; LF = low frequency; HF = high frequency

*p < .05

**p < .01

## Discussion

The present study is the first to investigate ER, autonomic and emotional reactivity, and autonomic and emotional recovery in SSD in response to sadness-induction. As expected, participants with SSD used maladaptive ER strategies more frequently and adaptive ER strategies less frequently than non-SSD participants (Hypothesis 1 supported). Yet, the groups did not differ in resting HRV (Hypothesis 2 not supported). Regarding reactions to the emotion induction, we only found different HRV activity patterns for lnSDNN in the form of an increase from baseline to recovery. For emotional reactivity and recovery, the groups only showed differences in energy levels: in the SSD group, the energy level decreased even during the recovery period (Hypotheses 3 and 4 partially supported). Concerning hypotheses 5, reduced vagal control was associated with higher somatic symptom severity but not with psychobehavioral aspects of SSD, depression or anxiety.

Our study dovetails with previous work on self-reported ER [5, 6], indicating ER deficits in persons with SSD. Two recent reviews [5, 6] conclude that persons with SSD use more suppression and less acceptance than healthy controls. This aligns with the results of our study in which persons with SSD also reported significantly more rumination and expressive suppression and less reappraisal and acceptance than healthy subjects. Accordingly, these results showed that the SSD group had a different use of habitual strategies than healthy subjects and therefore may have lacked appropriate ER strategy selection.

We did not find differences in HRV at rest between persons with and without SSD. This finding contrasts with previous work reporting lower HRV in persons with SSD [23–25] but aligns with other studies that found no abnormalities in HRV [48, 49]. Thus, the findings on HRV in SSD remain inconsistent, even though the recently published meta-analysis points to lower levels of HRV in SSD [22]. The HRV values in our study were higher than in previous studies on SSD [23–25, 30, 31]. Other sample characteristics, such as symptom burden, comorbidity, and illness duration were comparable to previous studies [25, 30]. Further potential influencing factors, such as individual differences (e.g., age, gender, BMI, medication) and situational factors (e.g., room temperature) were carefully controlled for and comparable in both groups. Thus, further research into moderators of the HRV – SSD association is needed to clarify the current heterogeneity in findings.

Regarding responses during emotion induction, we found no clear evidence for altered HRV reactivity or recovery in SSD. Interestingly, both groups also did not differ in their self-reported emotional reactivity and recovery. This finding is in line with some previous work [24] but contrasts with the conclusion of the recent meta-analysis on HRV in SSD [22]. This may be due to methodological differences between our and previous research. Most studies included in the meta-analysis used physical stressors or mentally challenging tasks. Our approach focused on evoking negative affect. It is possible that altered HRV reactivity and recovery would occur in reaction to symptom-related stimuli [30] and physical or mentally challenging tasks [22]. Moreover, the film sequences in the present study were shorter (two to three minutes) than emotion induction paradigms in previous research and only referred to one emotion (sadness). In future it is necessary to examine HRV reactivity in combination with other (negative) emotions. Future studies may benefit from using autobiographical memories as emotion induction as these have a stronger personal relevance and may produce longer-lasting reactions. Future research could also use longitudinal HRV measures (e.g., 24 h) combined with ecological momentary assessments to evaluate autonomic activity to stressors in daily life.

Our analyses revealed differential reactivity and recovery in lnSDNN between persons with and without SSD. This result aligns with a previous study on SSD [24]. According to the Model of Neurovisceral Integration, HRV increase is associated with ER strategy implementation. Therefore the increase of lnSDNN in the present study could reflect the increased use of ER strategies, e.g., rumination in the SSD group [50]. In that sense, the decrease in the energy level in the SSD group from baseline to recovery might reflect a higher effort to regulate emotions (see [13]). This higher effort could be explained by the observation that the SSD group used less adaptive ER strategies (e.g., rumination) than the non-SSD group.

Besides the conclusions based on group comparisons and within-group changes, our correlational analyses provided further insights into the association between HRV and SSD. For example, the link of symptom burden with lower HRV indicates that altered HRV could be a potential indicator of the severity psychopathology in SSD [19].

Finally, this study has noteworthy strengths and weaknesses. This is the first study assessing autonomic reactivity and recovery after emotion induction in participants diagnosed with SSD according to the DSM-5 criteria. We used a clinical interview and matched control group, which attests to the validity of our approach. Moreover, controlling for various potential confounders and thorough HRV artifact correction resulted in a good HRV data quality. There were also several limitations of the study. First, the sample size limits statistical power. Post-hoc power analyses (*α* = 0.05) showed that statistical power for a small effect was 1- *β* = 0.50 and for a medium effect 1- *β* = 0.99. Some previous studies in the research field showed comparable sample sizes (e.g. [23, 24, 30]). Unfortunately, we could not control for sex differences due to the sample and large proportion of female participants. However, sensitivity analyses did not provide evidence for a potential impact of gender on HRV results. As previous research suggests different HRV reactivity patterns in women and men, future studies with large and diverse samples are needed. Almost half of the participants in the SSD group reported at least one comorbidity, which is typical for SSD [51], but highlights the need to differentiate between mechanisms that are unique to SSD in future research.

## Conclusion

In sum, this study demonstrated ER deficits in persons with SSD. Thus, it appears recommendable to include ER techniques in interventions for SSD. For instance, the intervention program ENCERT included ER training in cognitive-behavioral therapy for SSD which resulted in better ER after therapy [52]. Also, HRV biofeedback improved ER skills and HRV in patients with SSD [34]. Yet, this study found only inconsistent evidence for an association of altered HRV or emotional reactivity and recovery with SSD. Therefore, the link of SSD with HRV requires further research using larger samples and different paradigms.

## Electronic Supplementary Material

Below is the link to the electronic supplementary material.

Supplementary Material 1
