# Supplementary material for: Heart Rate Variability, Autonomic Reactivity, and Emotion Regulation during Sadness Induction in Somatic Symptom Disorder
Source: Int J Behav Med. 2023 Oct 31;32(2):227–37. doi: 10.1007/s12529-023-10238-2 (PMC12031997; doi:10.1007/s12529-023-10238-2)
Supplement: Supplementary file 1 — Supplementary Material 1 [file 12529_2023_10238_MOESM1_ESM.docx]

**Table S1**

*Bivariate correlations (Spearman) between HRV parameters at rest and control variables variables for each group separately excluding individuals with cardiovascular medication*

| Variable | Group | Age | Gender | Tobacco consumption | Medical condition |
| --- | --- | --- | --- | --- | --- |
| lnSDNN | SSD | -.58** | -.05 | -.32* | -.28 |
|  | Non-SSD | -.52** | -.11 | .04 | -.17 |
| lnRMSSD | SSD | -.60** | -.15 | -.26 | -.20 |
|  | Non-SSD | -.47** | -.07 | .01 | -.19 |
| lnLF | SSD | -.48** | .15 | -.27 | -.31 |
|  | Non-SSD | -.49** | .07 | -.16 | -.15 |
| lnHF | SSD | -.55** | -.19 | -.30 | -.16 |
|  | Non-SSD | -.52** | -.13 | .10 | -.15 |

*Note.* SSD = somatic symptom disorder; ln = natural logarithm; SDNN = standard deviation of the NN interval; RMSSD = root mean square of successive differences; LF = low frequency; HF = high frequency.

**p* < .05

***p* < .01

|  | SSD (*n*=43) | |  | Non-SSD (*n*=41) | |
| --- | --- | --- | --- | --- | --- |
|  | *N* | *%* |  | *N* | *%* |
| **Medical condition** |  |  |  |  |  |
| hypertension | 3 | 0.07 |  | 3 | 0.07 |
| atrial fibrillation | 0 | 0 |  | 1 | 0.02 |
| kidney disease | 0 | 0 |  | 2 | 0.05 |
| rheumatism | 1 | 0.02 |  | 0 | 0 |
| asthma | 0 | 0 |  | 2 | 0.05 |
| cancer in the past | 0 | 0 |  | 2 | 0.05 |
| sleep apnoea | 0 | 0 |  | 1 | 0.02 |
| migraine | 1 | 0.02 |  | 1 | 0.02 |
| psoriasis | 0 | 0 |  | 2 | 0.05 |
| tinnitus | 0 | 0 |  | 2 | 0 |
| Hashimoto | 1 | 0.02 |  | 0 | 0 |
| POCS | 1 | 0.02 |  | 0 | 0 |
| craniomandibular dysfunction | 1 | 0.02 |  | 0 | 0 |
| morbus menière | 1 | 0.02 |  | 0 | 0 |
| **Medication** |  |  |  |  |  |
| beta-blocker | 2 | 0.05 |  | 0 | 0 |
| ace-inhibitors | 1 | 0.02 |  | 2 | 0.05 |
| statins | 1 | 0.02 |  | 1 | 0.02 |
| psychopharmaceuticals | 8 | 0.19 |  | 1 | 0.02 |
| contraceptives | 5 | 0.12 |  | 4 | 0.10 |
| pain medication | 16 | 0.37 |  | 3 | 0.07 |
| opioids | 2 | 0.05 |  | 0 | 0 |
| L-thyroxin | 8 | 0.19 |  | 1 | 0.02 |

**Table S2**

*Overview of the medical conditions and medications for persons with and without Somatic Symptom Disorder*

**Figure S1**

*HRV at baseline, during the emotion induction and the recovery period for the SSD and non- SSD groups*

A B


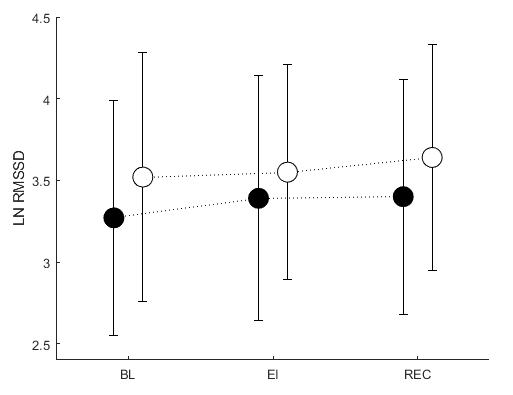

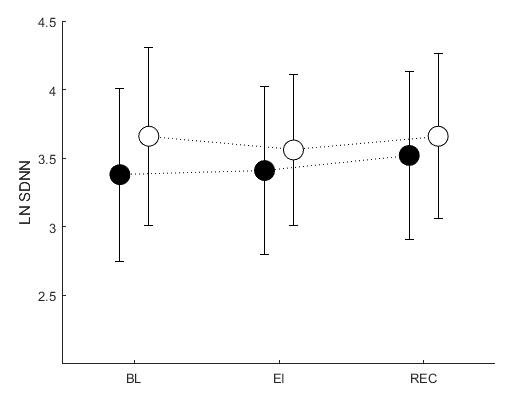


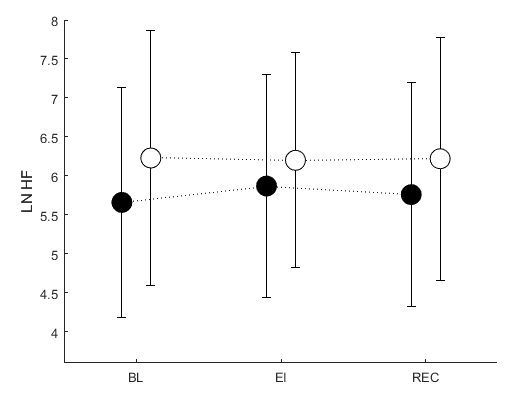
C D


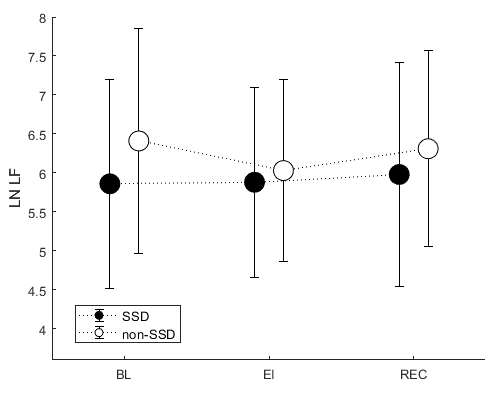


*Note*. A: Results for SDNN (mean values and standard deviations). B: Results for RMSSD (mean values and standard deviations). C: Results for LF (mean values and standard deviations). D: Results for HF (mean values and standard deviations). SSD = somatic symptom disorder; ln = natural logarithm; SDNN = standard deviation of the NN interval; RMSSD = root mean square of successive differences; LF = low frequency; HF = high frequency.

**Table S3***HRV at baseline, during the emotion induction and the recovery period for the SSD and non- SSD groups excluding male participants*

|  | SSD (*n*=32) | |  | Non-SSD (*n*=30) | |  |
| --- | --- | --- | --- | --- | --- | --- |
| Variable | *M* | *SD* |  | *M* | *SD* | ANOVA test statistics |
| **lnRMSSD** |  |  |  |  |  |  |
| BL | 3.33 | 0.74 |  | 3.58 | 0.84 | *F*_t_(2, 118) = 0.01, *p* = .99, *η_p_²*  = .00  *F*_g_(1, 59) = 1.05, *p* = .31, *η_p_²* = .02  *F*_int_(2, 118) = 2.01, *p* = .14, *η_p_²* = .03 |
| EI | 3.47 | 0.77 |  | 3.61 | 0.71 |  |
| REC | 3.44 | 0.75 |  | 3.58 | 0.76 |  |
| **lnSDNN** |  |  |  |  |  |  |
| BL | 3.39 | 0.65 |  | 3.70 | 0.71 | *F*_t_(2, 118) = 1.67, *p* = .19, *η_p_²* = .03  *F*_g_(1, 59) = 2.54, *p* = .12, *η_p_²* = .04  *F*_int_(2, 118) = 2.79, *p* = .07, *η_p_²* = .05 |
| EI | 3.45 | 0.62 |  | 3.60 | 0.62 |  |
| REC | 3.50 | 0.63 |  | 3.69 | 0.66 |  |
| **lnLF** |  |  |  |  |  |  |
| BL | 5.81 | 1.37 |  | 6.39 | 1.56 | *F*_t_(2, 118) = 1.97, *p* = .24, *η_p_²* = .03  *F*_g_(1, 59) = 1.66, *p* = .20, *η_p_²* = .03  *F*_int_(2, 118) = 2.06, *p* = .13, *η_p_²* = .03 |
| EI | 5.90 | 1.22 |  | 5.98 | 1.32 |  |
| REC | 5.92 | 1.25 |  | 6.33 | 1.36 |  |
| **lnHF** |  |  |  |  |  |  |
| BL | 5.83 | 1.51 |  | 6.37 | 1.80 | *F*_t_(1.73, 102) = 0.79, *p* = .44, *η_p_²* = .01  *F*_g_(1, 59) = 1.55, *p* = .22, *η_p_²* = .03  *F*_int_(1.73, 102) = 1.43, *p* = .25, *η_p_²* = .02 |
| EI | 6.04 | 1.50 |  | 6.31 | 1.49 |  |
| REC | 5.88 | 1.50 |  | 6.34 | 1.68 |  |

*Note.* SSD = somatic symptom disorder; HR = heart rate; ln = natural logarithm; RMSSD = root mean square of successive differences; SDNN = standard deviation of the NN interval; LF = low frequency; HF = high frequency; BL = baseline; EI = emotion induction; REC = recovery period.

**Figure S2**

*Subjective states at baseline, during the emotion induction and the recovery period for the SSD and non- SSD groups*

A B


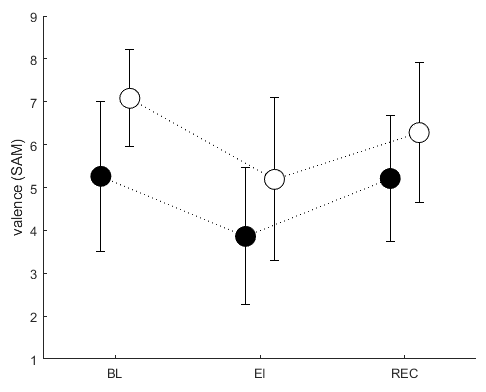

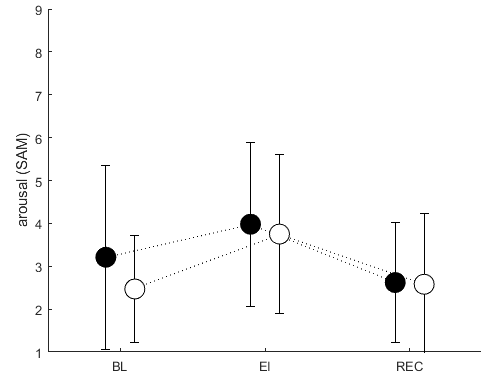


C D


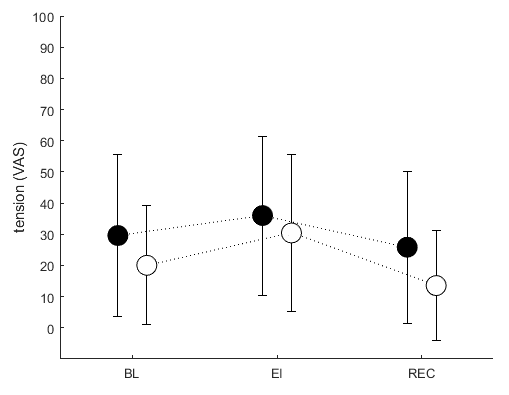

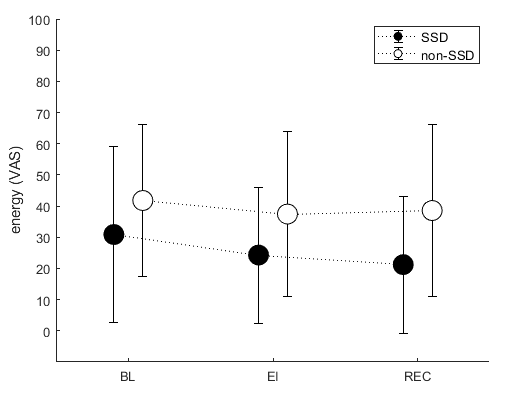


*Note*. A: Results for valence (mean values and standard deviations). B: Results for arousal (mean values and standard deviations). C: Results for tension (mean values and standard deviations). D: Results for energy (mean values and standard deviations). SSD = somatic symptom disorder; SAM = self-assessment manikin; VAS = visual analogue scale.

**Table S4**

*Subjective ratings (SAM and VAS) at baseline, in the emotion induction phase, and at the recovery period for the SSD and non-SSD groups excluding participants with heart medication*

|  | SSD (*n*=39) | |  | Non-SSD (*n*=37) | |  | |
| --- | --- | --- | --- | --- | --- | --- | --- |
|  | *M* | *SD* |  | *M* | *SD* | ANOVA test statistics | |
| **valence (SAM)** |  |  |  |  |  |  |  |
| BL | 5.21 | 1.81 |  | 7.03 | 1.17 | *F*_t_(1.62, 120) = 34.98, *p* < .001***, *η_p_²* = .32  *F*_g_(1, 74) = 23,52, *p* < .001***, *η_p_²* = .24  *F*_int_(1.62, 120) = 1.99, *p* = .15, *η_p_²* = .03 | |
| EI | 3.82 | 1.55 |  | 5.14 | 1.90 |  |  |
| REC | 5.23 | 1.53 |  | 6.24 | 1.62 |  |  |
| **arousal (SAM)** |  |  |  |  |  |  |  |
| BL | 3.11 | 2.12 |  | 2.57 | 1.24 | *F*_t_(2, 146) = 21.84, *p* < .001***, *η_p_²* = .23  *F*_g_(1, 73) = 0.24, *p* = .63, *η_p_²* = .00  *F*_int_(2, 146) = 1.37, *p* = .26, *η_p_²* = .18 | |
| EI | 3.89 | 1.93 |  | 3.86 | 1.87 |  |  |
| REC | 2.58 | 1.39 |  | 2.68 | 1.62 |  |  |
| **tension (VAS)** |  |  |  |  |  |  |  |
| BL | 29.64 | 26.32 |  | 20.13 | 18.82 | *F*_t_(2, 152) = 15.29, *p* < .001***, *η_p_²* = .17  *F*_g_(1, 76) = 2.89, *p* = .09, *η_p_²* = .04  *F*_int_(2, 152) = 1.44, *p* = .24, *η_p_²* = .02 | |
| EI | 33.92 | 25.77 |  | 31.18 | 25.12 |  |  |
| REC | 24.28 | 23.04 |  | 14.13 | 17.40 |  |  |
| **energy (VAS)** |  |  |  |  |  |  |  |
| BL | 30.00 | 28.93 |  | 40.46 | 24.34 | *F*_t_(2, 152) = 4.92, *p* = .009**, *η_p_²* = .06  *F*_g_(1, 76) = 6.65, *p* = .01*, *η_p_²* = .08  *F*_int_(2, 152) = 1.69, *p* = .188, *η_p_²* = .02 | |
| EI | 23.92 | 22.54 |  | 36.28 | 26.04 |  |  |
| REC | 20.41 | 22.76 |  | 38.15 | 27.24 |  |  |
| **sadness (VAS)** |  |  |  |  |  |  |  |
| BL | 17.49 | 22.63 |  | 5.62 | 7.50 | *F*_t_(1.58, 121) = 24.44, *p* < .001***, *η_p_²* = .24  *F*_g_(1, 76) = 2.90, *p* = .09, *η_p_²* = .04  *F*_int_(1.58, 121) = 1.59, *p* = .21, *η_p_²* = .02 | |
| EI | 35.49 | 31.05 |  | 29.49 | 27.10 |  |  |
| REC | 15.77 | 18.21 |  | 15.23 | 22.40 |  |  |

*Note.* SSD = somatic symptom disorder; VAS = visual analogue scale; SAM = self-assessment manikin; BL = baseline; EI = emotion induction; REC = recovery period.

* *p* < .05.

** *p* < .01.

*** *p* < .001.
